# 

**Published:** 1899-12

**Authors:** 


### The Library of the Bristol Medico-Chirurgical Society.

*The following donations have been received since the publication of the List in September:*

*November 30th, 1899.*

| | |
|---|---|
| Clifton Shakspere Society (1) ... ... ... ... | 1 volume. |
| Alban Doran (2) ... ... ... ... ... ... ... ... | 1 ,, |
| Sir William Mac Cormac, Bart. (3) ... ... ... | 1 ,, |
| Bertram M. H. Rogers, M.D. (4) ... ... ... | 75 volumes. |
| R. Shingleton Smith, M.D. (5) ... ... ... ... | 11 ,, |
| Surgeon-General, United States Army (6)... ... | 1 volume. |

Charles W. Sutton (7) ... ... ... ... ... ... ... 1 volume.
Government of the Colony of Tasmania (8) ... 2 volumes.
Council of University College, Bristol (9)... ... 1 volume.

Unbound periodicals have been received from Dr. R. Shingleton Smith.

---

### THIRTY-FOURTH LIST OF BOOKS.

The titles of books mentioned in previous lists are not repeated.

The figures in brackets refer to the figures after the names of the donors, and show by whom the volumes were presented. The books to which no such figures are attached have either been bought from the Library Fund or received through the *Journal.*

**Ashby and G. A. Wright, H.** *The Diseases of Children* ... ... 4th Ed. 1899
**Basham, W. R.** ... *Renal Diseases*... ... ... ... ... ... ... ... (4) 1870
**Bauhinus, C.** ... *Theatrum Anatomicum, accessit Appendix* ... ... ... 1600
**Beard and A. D. Rockwell, G. M.** *Medical and Surgical Electricity* ... (4) 1871
**Bennett, J. H.** ... *The Principles and Practice of Medicine* (4) 4th Ed. 1865
**Bentley, A. J. M.** *The Maintenance of Health in Egypt* ... ... 3rd Ed. [N.D.]
**Boott, F.** ... ... *Memoir of John Armstrong: to which is added an Inquiry into the Facts connected with Malaria Fever.* 2 vols. ... ... ... ... ... (4) 1833–34
**Bottone, S. R.** ... *Radiography* ... ... ... ... ... ... ... ... ... 1898
**Bryant, T.** ... ... *On Villous Growths and the Common Affections of the Rectum* ... ... ... ... ... ... ... ... ... 1899
**Bull, T.** ... ... *The Sense Denied and Lost* ... ... ... ... ... (4) 1859
**Carless, W. Rose and A.** *A Manual of Surgery* ... ... ... ... 2nd Ed. 1899
**Casserius** *Placentinus*, **J.** *Tabulæ Anatomicæ* lxxiix. *D. Bucretius* xx. *supplevit* ... ... ... ... ... ... ... ... ... 1632
*Catalogue (Index) of the Library of the Surgeon-General's Office, United States Army.* 2nd Ser., Vol. IV. (D—Emulsions) (6) 1899
**Catlow, J. P.** ... *Principles of Æsthetic Medicine* ... ... ... ... (4) 1867
**Clarke, J. J.** ... *Orthopædic Surgery*... ... ... ... ... ... ... ... 1899
**Clever, W.** ... ... *The Flower of Phisicke* ... ... ... ... ... ... ... 1590
**Cohn, B.** ... ... *Klinik der embolischen Gefässkrankheiten* ... ... (4) 1860
**Columbus, R.** ... *De Re Anatomica libri* xv. ... ... ... ... ... ... 1572
**Cosgrave, E. M.** ... *Experimental Proofs of the Role of Alcohol* ... ... ... 1899
**Credland, W. R.**... *The Manchester Public Free Li varies* ... ... ... (7) 1899
**Davis, J. H.** ... *Parturition* ... ... ... ... ... ... ... (4) 2nd Ed. 1865
**Denis, P. S.** ... *Recherches expérimentales sur le Sang humain* ... (4) 1830
**Doran, A.** ... ... *Shakespeare and the Medical Society* ... ... ... (2) 1899
**Duns, J.** ... ... *Memoir of Sir J. Y. Simpson, Bart.* ... ... ... ... 1873
**Eccles, A. S.** ... *Difficult Digestion due to Displacements*... ... ... ... 1899
**Edinger, L.** ... ... *Anatomy of the Central Nervous System of Man* (Tr. from 5th German Edition by W. S. Hall) 1899
**Ellis, H.** ... ... *Psychology of Sex.* 2 vols. ... ... ... ... ... 1897–1900
*Encyclopædia Medica* (Ed. by C. Watson). Vols. I., II. ... ... ... ... 1899

| | | | |
|---|---|---|---|
| **Féré, C.** | *The Pathology of Emotions* (Tr. by R. Park) | | 1899 |
| **Fourier, C.** | *The Passions of the Human Soul* (Tr. by Rev. J. R. Morell) | (4) | 1852 |
| **Fox, W. T.** | *The Classification of Skin Diseases* | (4) | 1864 |
| **Gamgee, J.** | *The Cattle Plague* | (4) | 1866 |
| **Garnett, R.** | *Essays in Librarianship and Bibliography* | | 1899 |
| **Geoffroy-Saint-Hilaire, I.** | *Vie, Travaux et Doctrine scientifique d'Étienne Geoffroy-Saint-Hilaire* | (4) | 1847 |
| **Gerhardt, C.** | *Lehrbuch der Kinderkrankheiten* | (4) | 1861 |
| **Gillespie, A. L.** | *A Manual of Modern Gastric Methods* | | 1899 |
| **Gregory, G.** | *Lectures on the Eruptive Fevers* | (4) | 1843 |
| *Harrogate as a Health Resort* | | (5) | 1899 |
| **Hedley, W. S.** | *Therapeutic Electricity* | | 1899 |
| **Hickman, W.** | *Cancerous Disease of Bone* | (4) | 1865 |
| **Hippocrates, C.** | *Opera quae extant, Graece et Latine, scholiis illustrata à H. Mercuriali.* 2 vols. in 1 | | 1588 |
| **Hobhouse, E.** [Ed.] | *Health Abroad* | | 1899 |
| **Howard, T.** | *On the Teeth* | (4) 5th Ed. | 1853 |
| **Jamin, J.** | *Cours de Physique* (4) Tomes I., 3e Éd., 1871, II. III., 2e Éd. | | 1868–69 |
| **Kerbert, J. J.** | *Klinische Memoranda* | (4) | [1859] |
| **Kölliker, A.** | *Mikroskopische Anatomie* Bd. II. Erste hälfte | (4) | 1850 |
| *Leamington Spa, Royal: the Midland Health Resort* | | (5) | [N.D.] |
| **Lebert, H.** | *Handbuch der allgemeinen Pathologie und Therapie* | (4) | 1865 |
| **Liebig, J.** | *Animal Chemistry* (Ed. by W. Gregory) | (4) 2nd Ed. | 1843 |
| **Mac Cormac, H.** | *Consumption* | (4) 2nd Ed. | 1865 |
| **Mac Cormac, Sir W.** | *The Hunterian Oration, 1899* | (3) | 1899 |
| **Macpherson, J.** | *Mental Affections* | | 1899 |
| **Madge, H.** | *The Diseases of the Fœtus in Utero* | (4) | 1854 |
| **Marsh, Sir H.** | *Clinical Lectures* (Ed. by J. S. Hughes) | (4) | [1867] |
| **Mays, T. J.** | *Pulmonary Consumption* | (5) | 1891 |
| **Méric, H. de** | *Dictionnaire des Termes de Médecine. Français-Anglais* | | 1899 |
| **Middleton, G. S.** | *Clinical Records* | (4) | 1894 |
| **Miles, A.** | *Surgical Ward Work and Nursing* | 2nd Ed. | 1899 |
| **Monro, T. K.** | *Raynaud's Disease* | | 1899 |
| **Mullen, B.** | *Salford and the Inauguration of the Public Free Libraries Movement* | | 1899 |
| **Murray, W.** | *Rough Notes on Remedies* | 3rd Ed. | 1899 |
| **Neale, R.** | *The Medical Digest—Appendix, 1891–99* | | 1899 |
| **Newman, D.** | *Renal Cases* | | 1899 |
| *Pamphlets* | 7 vols. | (4) | [V.D.] |
| **Physicist** | *Human Nature.* Part II. | | 1899 |
| **Pick, T. P.** | *Surgery* | | 1899 |
| **Piorry, P.-A.** | *De la Percussion médiate* | (4) | 1828 |
| **Platt, J. E.** | *Fractures and Dislocations of the Upper Extremity* | | 1899 |
| *Portsmouth, Southsea and Neighbourhood, Guide to* | (Ed. by H. T. Lilley) | (5) | 1899 |
| **Quain, R.** | *On some Defects in General Education* | (4) | 1870 |
| **Radcliffe, C. B.** | *Epilepsy and other Convulsive Affections* | (4) 2nd Ed. | 1858 |
| **Rayer, P.** | *Traité des Maladies des Reins.* 3 vols. | (4) | 1839–41 |
| **Robinson, M.** | *A Guide to Urine Testing* | | 1899 |

**Rockwell, G. M. Beard and A. D.** *Medical and Surgical Electricity* ... (4) 1871
**Rose and A. Carless, W.** *A Manual of Surgery* ... ... ... ... ... 2nd Ed. 1899
**Rutlidge, C. S.** ... *Guide to Queensland* ... ... ... ... ... ... ... (1) [N.D.]
*Sanitary Institute, The* *Illustrated List of Exhibits to which Medals have been Awarded at their Exhibitions, 1889–98* ... 1899
**Seubert, M. C. W.** *De Functionibus radicum arteriorum et posteriorum Nervorum Spinalium Commentatio* ... ... (4) 1833
*Shaw's Manual of the Vaccination Law* ... ... ... ... ... ... ... 7th Ed. 1899
**Smith, F. J.**... ... *Differential Diagnosis with Clinical Memoranda* ... ... 1899
**Snell, S.** ... ... *On the Prevention of Eye Accidents Occurring in Trades* 1899
**Spencer, W.**... ... *Spencer's Disease* ... ... ... ... ... ... ... ... ... 1899
**Spigelius, A.** ... *De Humani Corporis Fabrica libri decem* ... ... ... 1632
**Steffen, A.** ... ... *Klinik der Kinderkrankheiten.* 2 vols.... ... (4) 1865–70
**Stephanus, N.** ... *Castigatio Epistolæ maledicæ quam L. de Bils scripsit ad T. Bartholinum* ... ... ... ... ... ... ... ... 1661
**Stonham, C.**... ... *A Manual of Surgery.* Vols. I., II. ... ... ... ... 1899
**Swieten, G. van** ... *The Commentaries upon the Aphorisms of Dr. H. Boerhaave* (Translated) (4) Vols. VI., 1757, IX. 1765
*Tasmania, Handbook of.* *Hints to Intending Settlers and Immigrants* (8) 1897
,, ,, *With list of Reference Works on the Resources of the Colony* ... ... ... ... ... ... ... ... ... (8) 1899
**Temple, W.**... ... *Pro Mildapetti de Unica Methodo Defensione contra Diplodophilum Commentatio* ... ... ... ... ... 1584
**Thomson, H. C.** ... *An Introduction to Diseases of the Nervous System* ... 1899
**Thorne, W. B.** ... *The Schott Methods of the Treatment of Chronic Diseases of the Heart* ... ... ... ... ... ... 3rd Ed. 1899
**Valleix, F.-L.-I.**... *Guide du Médecin Praticien* 5 vols. (4) 4e Éd. 1860–61
*Ventilation, Natural and Artificial Methods of*... ... ... ... ... ... ... ... 1899
**Vesalius, A.** ... *De Humani Corporis Fabrica libri septum* ... ... ... 1543
,, ... *Suorum de Humani Corporis Fabrica Librorum Epitome* 1600
**Virchow, R.** [Ed.] *Handbuch der speciellen Pathologie und Therapie* Bd. I. ... ... ... ... ... ... ... ... ... (4) 1854
**Walker, Jane H.** *Open-Air Treatment of Consumption* ... ... ... ... 1899
**Walshe, W. H.** ... *The Physical Diagnosis of Diseases of the Lungs*... (4) 1843
,, .. *Diseases of the Lungs and Heart* ... ... ... ... (4) 1851
**Watson, J. K.** ... *A Handbook for Nurses* ... .. ... ... ... ... ... ... 1899
**Whytt, R.** ... ... *Observations on those Disorders commonly called Nervous, Hypochondriac, or Hysteric* ... ... (4) 3rd Ed. 1867
**Wilson, E.** ... ... *Lectures on Dermatology*... ... ... ... ... ... (4) 1875
**Wooton, E.**... ... *A Guide to the Medical Profession* (Ed. by L. F. Winslow) ... ... ... ... ... ... ... ... (4) [1882]
**Wright, H. Ashby and G. A.** *The Diseases of Children* ... ... 4th Ed. 1899

### TRANSACTIONS, REPORTS, JOURNALS, &c.

Advertiser's A B C, The ... ... ... ... ... ... ... ... ... ... (4) 1893
American Public Health Association, Reports and Papers of the Vol. XXIII. 1898
Archives of Neurology from the Pathological Laboratory of the London County Asylums ... ... ... ... ... ... ... ... ... .. ... [N.D.]
Archives of the Roentgen Ray ... ... ... ... ... ... ... Vol. III. 1899

Columbia University, Studies from the Department of Pathology of the College of Physicians and Surgeons Vol. VI., Part 1 1898-99
Edinburgh Medical Journal ... ...Vols. XIV., Part 2, XV., Part 1 1869-70
Edinburgh Obstetrical Society, The Transactions of the Vol. XXIV. 1899
Epidemiological Society of London, Transactions of the N.S., Vol. XVIII. 1899
Hospitals—
Dublin Hospital Reports, The ... ... ... ...Vols. III.—V. 1822-30
Johns Hopkins Hospital Reports, The Vols. VII., Nos. 5-9, 1899; VIII., Nos. 1, 2 1899
London Hospital for Diseases of the Chest, City of—Pathological Report, 1887-88 ... ... ... ... ... ... ... ... .. (5) 1889
Hospitals and Charities, Burdett's ... ... ... ... ... ... ... ... 1899
Journal of Mental Science, The ... Vols. IV.—VI., 1858-60; VIII.—XII., 1863-67; XIV.—XVIII., 1869-73; XLII., XLIII., 1896-97; General Index Vols. I.—XXIV., 1879; XXV.—XXXVIII. [1893]
Medical Annual Synoptical Index, The, 1887-98 ... ... ... ... ...[1899]
Medical Society of London, Transactions of the ... ... Vol. XXII. 1899
Medico-Chirurgical Transactions .. Vols. LXXIX., 1896; LXXXII. 1899
Mexico—Informes Rendidos por los Inspectores Sanitarios de Cuartel y los de los Distritos al Consejo Superior de Salubridad, 1898... 1899
Michigan State Medical Society, Transactions of the ... Vol. XXIII. 1899
New York, Transactions of the Medical Society of the State of ... ... 1899
Ohio State Medical Society, Transactions of the ... ... ... ... ... 1899
University College, Bristol—Calendar for the Session 1899-1900 ... (9) 1899

---

### Local Medical Notes.

### BARNSTAPLE.

North Devon Infirmary.—Mr. Joseph Harper presided at the annual meeting of those interested in this institution on August 15th. The medical report showed that 622 patients had been admitted during 1898. The average daily number was 41 and the average stay was twenty-three days. The financial statement showed that the expenditure amounted to £2,362, which is much above the ordinary income of the infirmary. The friendly societies of Barnstaple have recently collected over £200 for the local Infirmary and Dispensary.

### BATH.

Sewerage.—A considerable amount of money has been spent upon the proposed sewage scheme for Bath. Land has been purchased at Salford, where it was proposed that the sewage should be intercepted and chemically treated. The Local Government Board have recently stated that they were not prepared to entertain any proposal as to a partial scheme for sewage disposal, and consequently for the present the matter is in abeyance. Apparently the only effective manner of disposing of the sewage of Bath without sending it into the river is for the authorities of that city to join in the Bristol scheme, and take the sewage through a large drain to the Bristol Channel. The Bristol Authority has approached the Bath Corporation upon the matter.

Public Health.—Dr. W. H. Symons has been re-appointed Medical Officer of Health for Bath, at a salary of £435 per annum.

### BRENTRY.

Royal Victoria Home.—Dr. W. Cotton has been appointed, *pro tem.*, Honorary Medical Officer.

### BRIDGWATER.

Public Health.—Mr. F. W. Stoddart has been re-appointed Borough Analyst.

### BRISTOL.

The Convalescent Home.—The visit of Her Majesty the Queen to open the Convalescent Home, which is for all time to maintain the memory of her sixty years of reign over her people, was, from the point of view of the many thousands who lined the route of procession, a small matter. What really interested the citizens of Bristol and the inhabitants of the neighbourhood was the presence of Her Majesty herself. This is hardly the place to enlarge on the loyalty of her subjects; but at the present time, when the Queen shows a greater interest, if possible, in the welfare of her people than she has ever shown, mention must be made of the enthusiastic reception she received from the moment she stepped out of the railway carriage to the time she entered it again. During the two hours she was in Bristol the roar of applause and cheers was continuous, and many a young child in years to come will remember the day when Queen Victoria was drawn by four horses through the streets of our ancient city. The weather, as it usually is when the Queen has any function to perform, was beautiful; in fact the day was just the one the Queen is said, and we believe truly, to like—crisp, sunny, and with a nip in the air, which does not please the fancy of many of her less robust subjects. At any rate, Sir Arthur Bigge telegraphed soon after Her Majesty's return to Windsor that not only was she much moved by her loyal reception by the city of Bristol, but that she was not unduly fatigued by the journey. When we consider Her Majesty's advanced age, many will wonder how she is able to bear, in the marvellous manner she does, the ordeals of such a reception and the fatigue of a journey of nearly two hundred miles by rail.

Of the civic ceremonies which took place at the Joint Station and the Council House little mention need be made in a medical periodical, except to record the graceful action of Her Majesty in dubbing the Lord Mayor a Knight, an honour which was exceedingly popular. At the Convalescent Home, Sir Herbert Ashman presented Sir Edward P. Wills, upon whom a K.C.B. has been recently so deservedly bestowed in recognition of his work in connection with the Institution. After a prayer from the Bishop of Bristol, and the singing of the hymn "Oh, King of kings" to the tune "Bishopgarth," an address was read to Her Majesty, who returned a written reply, and the President presented his daughter (from whom the Queen accepted a bouquet), Mr. J. Storrs Fry, Mr. Edward Robinson, Mr. P. H. Vaughan, the Rev. R. Glover, D.D., Mr. J. N. C. Pope, Mr. H. O. Wills, Sir Frederick Wills, Bart., Mr. F. J. Fry, Mr. Charles Thomas, Mr. P. F. Sparke Evans, Mr. W. L. Bernard, and the Matron (Miss Ellis).

The Queen's carriage was then drawn to the front entrance, and with an electric communication Her Majesty opened the door, the "jewelled electrical letter-weight" with which this part of the ceremony was performed being presented to her as a *souvenir.*

The procession then reformed and passed onwards to the station, which Her Majesty left soon after 4 p.m.

Reference must be made to the monster gathering of children on Durdham Down. Here, in a stand about a third of a mile in length, about 27,000 children were assembled, and, under the conductorship of Mr. Riseley, sang to Her Majesty two verses of the National Anthem. Those who were near inform us that the Queen was deeply touched at the sight, and the sounds of so many young voices. The shrill cheering could be heard a mile away. Since the ceremony, Her Majesty has telegraphed to enquire whether the children arrived safely at their homes, and it is a matter for sincere congratulation to those who so ably organised this wonderful gathering that not only was no child lost in the crowds, but that there was no accident of any sort, and among the immense number there were only four cases of fainting. Indeed, from what we can hear, the amount of work which the voluntary members of the St. John Ambulance Association had to do was inconsiderable. No serious accident has been reported, except one at St. Philip's Station late at night, when a lady was pushed under a train with fatal results. When we hear that the crowd has been calculated to have been composed of 700,000 persons, this speaks well for the good humour and patience of everyone.

Medical School Dinner.—The revival of the Medical School dinner reflects great credit on the organisers. It is now some years since the annual dinner was discontinued, and, if we can judge from the numbers who assembled at the Spa on November 16th, the new lease of life appears to be vigorous. Mr. Nelson C. Dobson took the chair, having on his right the guest of the evening, His Honour Judge Austin, and near them were seated most of the Professors of Bristol University College, both those connected with the Faculty of Medicine and of Arts. After dinner, the Chairman proposed in excellent taste the health of Her Majesty, and announced that a telegram had been sent to Windsor to say how gratified those assembled were to hear of Her Majesty's safe journey and that she was not over-fatigued by the ceremony of the 15th. The speech, however, of the evening was that made by the Rev. A. N. Blatchford in proposing "The Army, Navy, and Reserve Forces," which at a time like the present is sure to find a hearty response. Everyone—to use Kipling's expression —shouted "Rule Britannia" at the top of their voices, for Mr. Blatchford's oratory touched the patriotic *chordæ tendineæ.* Prof. Ryan, whose departure from Bristol at an early date robs us of our most amusing after-dinner speaker, indulged in his usual pleasantries, and was well received, while other speeches were made by Judge Austin, Dr. J. O. Symes, Mr. A. W. Prichard, Dr. A. J. Harrison, Mr. A. L. Flemming, Mr. Munro Smith, Dr. W. H. Daw of H.M.S. *Antelope,* and Mr. F. R. Cross. The Chairman's health was proposed by Dr. Shingleton Smith in felicitous terms, and Mr. Dobson replied. The gaiety of the evening was increased by several excellently-rendered songs, and the only criticism of an adverse nature we have to make was that the proceedings were rather too long, that a suspicion of rowdiness was noticed in one region of the room, and that the room was rather cold. We have no doubt that the School dinner will be an annual institution.

The point to which most speakers referred was the recent amalgamation of the clinical work of the Infirmary and Hospital, and the position of the Medical Faculty with regard to the University College, some claiming that the Medical School, as the elder, was the parent of the College, while others, though admitting the age of the Medical Department, argued that the University College was its mother. The physiological impossibility of the daughter being older than the mother did not seem to trouble those who claimed a maternal position for the Bristol University College.

### CARDIFF.

Prevention of Consumption.—At a well-attended meeting of the Cardiff Medical Society, held on October 13th, a resolution was unanimously carried approving of the formation of a local branch of the National Association for the Prevention of Tuberculosis. The Medical Officers of Health of Cardiff and Rhondda, in supporting the resolution, asked for the assistance of individual members of the medical society in addressing public meetings in Glamorgan and the adjoining counties. The Medical Officer of Health of Cardiff (Dr. Edward Walford) is issuing leaflets in connection with the steps being taken for the prevention of tuberculosis by the sanitary authority. In one of these it is stated that in Cardiff, out of a total number of about 2,500 deaths which occur annually from all causes, about 200 are due to phthisis. Instructions are given as to the precautions necesssary to prevent the spread of infection from a consumptive person to those about him. In another leaflet issued to dairymen, cowkeepers, and purveyors of milk, instructions are given as to the means of preventing the spread of tuberculosis among cattle.

Infirmary.—Dr. W. M. Stevens has been appointed Honorary Pathologist.

### CHELTENHAM.

Obituary.—Mr. Lauriston Winterbotham died at his residence, Arundel House, on August 8th. The deceased, who was in his sixty-sixth year, was the son of the late Mr. John Winterbotham, and his brother, Mr. Alderman J. B. Winterbotham, is the deputy-mayor of Cheltenham. Mr. Winterbotham received his medical education at St. Bartholomew's Hospital, and qualified as M.R.C.S. Eng. and L.S.A. in 1858. He shortly afterwards commenced practice in Cheltenham, and was for several years surgeon to the General Hospital; upon his resigning this appointment, he was elected consulting surgeon to the institution. He was also consulting surgeon to the Dispensary for Diseases of Women and Children, and surgeon to the Cheltenham College. Mr. Winterbotham (who married a daughter of the late Mr. Solomon Leonard, of Clifton) has left a widow and three children to mourn his loss. The funeral took place on August 11th, at the Cheltenham Cemetery, in the presence of a large number of people.

### CHEPSTOW.

Obituary.—Mr. Edward Pendrill King died on September 23rd, in his 66th year. He received his medical education at St. Bartholomew's Hospital, and took the qualifications of M.R.C.S. Eng. and L.S.A. in 1858 and 1859 respectively. The deceased held several public appointments, being formerly coroner for the manor of Chepstow, medical officer of health of Chepstow, and also medical officer of the Shirenewton District and the workhouse of the Chepstow Union. Mr. King was assistant-surgeon to the 1st Monmouthshire Rifle Volunteers.

He had been in failing health for some months past, and had recently resigned all his appointments. Mr. King will be much missed in Chepstow, where he was extremely popular.

### DAWLISH.

Hospital.—The recent fête organised on behalf of the Hospital has resulted in a sum of £236 being handed over to that institution.

### DEVONPORT.

Royal Albert Hospital.—Mr. J. J. N. Morris has been appointed Assistant Surgeon.

### DORCHESTER.

Lunacy.—At the quarterly meeting of the Dorset County Council, held on August 1st, Lord Digby, the chairman of the Asylums Committee, referred to the great increase of pauper lunacy in the county, and stated that additional accommodation would shortly have to be provided, although the new asylum had only recently been opened.

### EXETER.

Typhoid Fever.—At the meeting of the City Council, held on September 13th, it was stated that in the recent outbreak of typhoid fever at Exeter seventy out of eighty cases had been traced to the eating of uncooked cockles at Exmouth. The city analyst had examined the effluent from the mouth of the sewer at Exmouth, where the cockles were gathered, and he had found that it consisted of a mixture of sea-water and untreated sewage. It was stated that the sale of the cockles had been stopped at Exmouth, but that they were being sent to other towns. Mr. E. J. Domville proposed that the Devon County Council should be communicated with, in order that an application might be made to the Local Government Board to declare the whole of the estuary of the river Exe a stream within the meaning of the Rivers Pollution Act. After some discussion, the motion was carried. At the meeting of the City Council held on September 27th, a special report was presented by the senior District Medical Officer of the city upon the recent epidemic of typhoid fever. This stated that from July 1st to September 11th there were 85 cases of the disease notified, and of these 58 patients had partaken of raw cockles at or from Exmouth. Nine of the patients had only visited Exmouth, and one had partaken of other shell-fish than cockles. 67 cases had been treated at the sanatorium, with five deaths. One of the nurses there had contracted the disease. The cost of the epidemic would be between £1,500 and £2,000. During the discussion which followed the reading of the report some of the members said that they ought to be grateful to their medical officers for the steps which they had taken to cut short the epidemic.

West of England Eye Infirmary.—The annual meeting of this institution was held on October 27th. The medical report stated that 2,891 patients had been under treatment, of whom 2,517 had been discharged cured and relieved, so that on September 30th there remained 333 out-patients and 41 in-patients. The daily average number of in-patients had been 46. The financial statement showed that the ordinary income amounted to £1,494, and that there was a favourable balance of £179. The committee added that satisfactory progress is being made with the first section of the new building, which will be shortly completed, and an earnest appeal was made for £9,000 to continue the work.

### FALMOUTH.

Typhoid Fever.—At the meeting of the Falmouth Corporation held on September 28th, the memorandum issued by Dr. G. S. Buchanan (of the Local Government Board) was considered. Dr. Buchanan advises that the residents should be cautioned to boil all water, and that the sewers should be flushed. He adds that there is a large number of defective house drains in Falmouth, and advises that all should be thoroughly supervised, and recommends the appointment of a second Sanitary Inspector without delay. The Corporation decided to follow the advice of Dr. Buchanan, who is still pursuing his enquiries into the sanitary condition of the town.

### HALWELL.

A Munificent Gift.—Mrs. Medley has promised to build, furnish, and endow a cottage hospital for Halwell, Devonshire. The building, which will contain four wards, is to be furnished by July next, the cost being about £2,220.

### KINGSBRIDGE.

Typhoid Fever.—At the meeting of the Urban District Council, held on October 4th, the Medical Officer of Health (Dr. Webb) reported that there had been three cases of typhoid fever caused by eating cockles.

### MAESTEG.

Memorial to a Medical Man.—The fund for providing a memorial to the late Dr. W. Hopkin Thomas has just been closed. The sum realised was about £700, and it has been decided that the memorial should take the form of a fountain with commemorative tablets.

### PENRYN.

Typhoid Fever.—At the meeting of the East Kerrier Rural District Council, held on August 5th, the Medical Officer of Health reported that he had traced a case of typhoid fever to the patient having eaten cockles gathered from the mud of Penryn river. Last year he had traced three cases to the same cause.

### PLYMOUTH.

Public Dispensary.—The annual meeting of those interested in the Plymouth Public Dispensary was held on October 18th. The report showed that during the year 3,670 patients had been treated, against 3,345 in the preceding year. In the provident department there were 112 admissions during the year—an increase of 29—and there were at present 696 names on the books. The expenditure amounted to £1,070, as against £987 for 1898. The gross receipts of the provident department were £132, against £128 in the previous year. The annual subscriptions were £367, as compared with £359 in 1898. The income from dividends amounted to £421, and Sir Massey Lopes had contributed £1,000 during the past two years. It has been decided to extend the accommodation of the Dispensary, and new buildings were being erected at a cost of £575. The chairman urged that this being the centenary year of the institution a strong appeal should be made to wipe off the debt of £219.

### ST. GERMANS.

Vaccination.—At the meeting of the Board of Guardians, held on August 2nd, it was stated that the vaccination fees for the past six months were four times the amount of those for the preceding half-year.

### STROUD.

Infectious Disease Hospital.—It is stated that the Stroud Joint Hospital Board have decided upon a site in the populous district known as Horns Road, for the erection of an infectious disease hospital. This proposal has already met with some opposition on the part of ratepayers.

### SWANSEA.

General Hospital.—Every alternate year for seventeen years past the Baroness Patti-Cederström (Madame Patti) has organised a concert in aid of the General Hospital and other charities in the town. Not only does she herself take part in the programme, but she induces other well-known artistes to volunteer their services, entertaining them at Craig-y-nos and bringing them by special train to Swansea. The last of this series of concerts was given on August 3rd, and is considered to have quite eclipsed any of its predecessors. About £600 was realised. Dr. D. E. Evans has been appointed Anæsthetist and Pathologist.

### TAUNTON.

Sewerage.—At a special meeting of the Town Council, held on September 7th, it was decided to adopt the septic tank system of sewage disposal. The cost was estimated at about £22,000.

Taunton and Somerset Hospital.—£1,000 have been subscribed in Somerset as a memorial to the late Colonel Chard, V.C. It has been decided to erect a memorial bust and to endow a bed in the Taunton and Somerset Hospital for a Somersetshire-born soldier, his wife, widow, or child.

### TAVISTOCK.

Public Health.—Mr. E. C. Brodrick has been re-appointed Medical Officer of Health.

### THORNBURY.

Public Health.—Dr. F. T. Bond has been re-appointed Medical Officer of Health.

### TIVERTON.

Infirmary.—The new wing, which has been erected to celebrate Her Majesty's Diamond Jubilee, was formally opened by Sir Stafford Northcote, M.P., on August 10th, in presence of a large gathering. The new building comprises two wards of two beds each for male and female patients, an operation-room, nurses' room, waiting-room, etc. The cost was about £1,550.

### TORQUAY.

Incipient Consumption Hospital.—On the suggestion of the honorary medical staff, the committee of the Western Hospital for Incipient Consumption, some few months ago, made a special appeal to the public for funds, with the result that the hospital has been fitted with balconies and wind-screens for the adoption of the open-air treatment, the ventilation of the building has been improved, and the electric light installed.

OCKERDEN CONVALESCENT HOME.—Lady Helen Vincent re-opened this institution at Torquay on November 2nd. The Home was founded in 1890 by Miss M. Erle, who died last year, and it has now been given by her sister, Miss Eleanor Erle, for the use of patients from the Royal Devon and Exeter Hospital. With the gift of the Home, Miss Erle gave £400 to cover the cost of completely renovating the building.

### TRURO.

ROYAL CORNWALL INFIRMARY.—The annual meeting of the friends of this institution was held on August 14th. The annual report showed that during 1898 there had been 416 in-patients admitted, as against 369 in the preceding year. The average duration of residence was 27 days, against 31 days in 1897. The average daily number of in-patients was 31. The out-patients numbered 650, an increase of 149 compared with the preceding year. The receipts amounted to about £2,100, as against £1,858 in 1897. The expenditure was £1,900, being nearly £100 more than last year.

### WESTBURY.

PUBLIC HEALTH.—Mr. W. H. Reed has been appointed Medical Officer of Health.

### YEOVIL.

WATER SUPPLY.—The new reservoir, constructed to hold 1,000,000 gallons, is now completed.

### The College of Physicians of Philadelphia.

### LIST OF SUBSCRIBERS

TO

### The Bristol Medico-Chirurgical Journal.

*DECEMBER*, 1899.

---

### England.

### CAMBRIDGESHIRE:

CAMBRIDGE.
G. S. Woodhead

### CHESHIRE.

ALDERLEY EDGE.
J. McElfatrick

NEW BRIGHTON.
A. W. Riddell

### CORNWALL.

EAST LOOE.
L. F. Houghton

HELSTON.
B. C. Kendall

LISKEARD.
S. E. Rigg

PENZANCE.
W. S. Bennett
J. A. Fox
J. Symons

ST. AUSTELL.
W. Mason

WEEK-ST-MARY.
S. H. Rentzsch

### DEVONSHIRE.

BABBACOMBE.
T. Finch

BAMPTON.
A. R. Down

BARNSTAPLE.
C. H. Gamble
J. Harper
M. Jackson

BRIXHAM.
G. C. Searle

BROAD CLYST.
J. Somer

BUDLEIGH SALTERTON.
T. G. C. Evans

CHAGFORD.
A. D. Hunt

CULLOMPTON.
G. G. Gidley

DARTMOUTH.
R. B. Searle

DEVONPORT.
F. E. Row

EXETER.
J. M. Ackland
H. Davy
R. V. Solly
E. B. Steele-Perkins
R. Thomas
L. H. Tosswill

NEWTON ABBOT.
J. Culross

PAIGNTON.
J. Alexander

PLYMOUTH.
G. F. Aldous
R. H. Clay
Medical Society
E. B. Thomson
W. L. Woollcombe

PLYMPTON.
C. Aldridge
W. D Stamp

### DEVONSHIRE *(Continued)*.

SOUTH MOLTON.

H. J. Smyth

TORQUAY.

H. Humphreys
W. Odell
R. Pollard
W. Powell
C. H. Wade

WINKLEIGH.

J. H. Norman

### DORSETSHIRE.

DORCHESTER.

P. W. MacDonald

STURMINSTER NEWTON.

J. C. Leach

WEYMOUTH.

J. M. Lawrie

### ESSEX.

NEWPORT.

W. A. Smith

### GLOUCESTERSHIRE.

ALMONDSBURY.

J. C. MacWatters

BRISTOL.

W. R. Ackland
J. Ambrose
T. R. Atkinson
W. M. Barclay
T. G. L. Baretti
B. J. Baron
A. E. Blacker
A. F. Blagg
E. C. Board
C. W. J. Brasher
H. F. Briggs
J. Broom
F. St. J. Bullen
E. F. H. Burroughs
J. P. Bush
A. Carr
T. M. Carter
W. F. Carter
T. Carwardine
R. H. Chilton
J. M. Clarke
T. V. Coker
H. Cook
F. N. Cookson
W. Cotton
R. J. Coulter
F. R. Cross
J. Dacre
D. S. Davies
N. C. Dobson
E. A. G. Dowling
E. L. W. Dunbar
E. Eberle
F. H. Edgeworth
W. Elder
C. Elliott
J. Ewens
E. Fawcett
R. G. Fendick
J. L. Firth
T. Fisher
A. L. Flemming
E. L. Fox
J. Freeman
W. J. Fyffe
E. B. Garland
T. T. Genge
W. C. Gent
D. S. Gerrish
A. N. G. Gibbs
J. S. Griffiths
L. M. Griffiths
W. B. T. Gubbin
C. D. G. Hailes
A. H. Haines
H. E. Harris
A. J. Harrison
W. H. Harsant
A. G. Hayman
C. A. Hayman
J. C. Heaven
C. K. C. Herapath
H. E. Hetling
H. Hill
T. Hill
General Hospital
G. A. Imlay
H. W. Kendall
F. P. Lansdown
R. G. P. Lansdown
A. E. A. Lawrence
E. L. Lees
R. C. Leonard
W. Ligertwood
J. J. S. Lucas
P. W. McCrea
A. B. McKee
G. Metcalfe
W. A. Milligan
H. F. Mole
A. T. Morgan
C. A. Morton
G. T. Myles
B. G. Neale
W. N. Nevill
C. Newman
W. H. C. Newnham
T. D. Nicholson
C. O'Brien
A. Ogilvy
G. S. Page
G. Parker
J. H. Parry
A. W. Peake
H. C. Pearson
W. A. Perry
C. F. Pickering
W. E. Pountney
J. J. Powell
A. W. Prichard
J. E. Prichard
A. B. Prowse
B. M. H. Rogers
F. Rose
W. B. Roué
C. K. Rudge
H. T. Rudge
J. E. Shaw
E. J. Sheppard
E. M. Skerritt
G. M. Smith
R. S. Smith
E. H. E. Stack
W. H. Steele
C. Steele
J. B. Stephenson
W. H. Stevens
F. W. Stoddart
J. Swain
J. G. Swayne
S. H. Swayne
W. C. Swayne
J. O. Symes
J. Taylor
W. J. Tivy
H. Waldo

### GLOUCESTERSHIRE *(Continued)*.

BRISTOL *(continued)*.

C. H. Walker
C. H. Wallace
J. H. Wathen
C. J. Whitby
J. Wilding
E. C. Williams
P. W. Williams
W. R. Williams
H. W. Windsor-Aubrey
C. Wintle

CHELTENHAM.

W. J. K. Millard
G. A. Peake
E. Trevithick
L. Winterbotham

CHIPPING SODBURY.

F. F. Fox
A. Grace

CIRENCESTER.

W. R. Cossham
O. H. Fowler
C. Mackinnon

COLEFORD.

L. B. Trotter

DOWNEND.

H. Skelton

FISHPONDS.

W. Brown

GLOUCESTER.

R. W. Batten
W. R. Hadwen
W. Hodges
J. G. Soutar

HAMBROOK.

E. Crossman
F. W. Crossman
W. F. B. Eadon

KINGSWOOD.

L. W. Powell

LYDNEY.

W. P. Kennedy

PARKEND.

W. C. Halpin

REDFIELD.

J. W. Rodgers

ST. GEORGE.

J. Young

STAPLETON.

H. A. Benham

STOKE BISHOP.

H. F. Parsons.
C. E. Wedmore

STONEHOUSE.

G. T. B. Watters

STOW-ON-THE-WOLD.

E. Dening

STROUD.

A. S. Cooke

TETBURY.

W. Wickham

THORNBURY.

E. M. Grace
L. H. Williams

WESTBURY-ON-TRYM.

A. Harvey
H. L. Ormerod

WINTERBOURNE.

R. Eager
W. Eager

WOTTON-UNDER-EDGE.

J. G. Boyce
D. H. Forty

### HAMPSHIRE.

ALTON.

W. L. P. Bevan

BOURNEMOUTH.

C. H. Ackland
J. S. Dickie
J. G. Harsant

CARISBROOKE.

J. Groves

FLEET.

H. Wilcox

LYMINGTON.

J. Rendall

SOUTHAMPTON.

W. R. Y. Ives

SOUTHSEA.

J. W. Cousins

### HUNTINGDONSHIRE.

ST. NEOT'S.

T. C. Grey

### LANCASHIRE.

LIVERPOOL.

G. A. Hawkins-Ambler

PRESTON.

J. E. Garner

### MIDDLESEX.

### LONDON.

Bayer Co.
J. Berry
T. C. Fox
M. Handfield-Jones
W H. A. Jacobson
H. M. Jones
Lord Lister
E. J. Maclean
J. Macready
A. L. Marshall
F. T. Roberts
W. J. Seward
E. C. Taylor

### TOTTENHAM.

W. H. Plaister

### MONMOUTHSHIRE.

### NEWPORT.

W. Basset
C. B. Gratte
O. E. B. Marsh
C. Matthews
A. G. Thomas
H. E. Williams

### PONTYPOOL.

J. R. Essex
S. B. Mason

### RHYMNEY.

T. H. Redwood

### TREDEGAR.

G. A. Brown

### NORFOLK

### WALPOLE ST. ANDREWS.

R. Smith

### NOTTINGHAMSHIRE.

### NOTTINGHAM.

C. B. Taylor
W. M. Willis

### OXFORDSHIRE.

### OXFORD.

H. P. Symonds

### SOMERSETSHIRE

### BATH.

W. D. Akers
E. J. Cave
F. S. Cowan
S. Craddock
W. M. Ellis
A. E. W. Fox
T. B. Goss
P. King
F. Lace
T. P. Lowe
W. S. Melsome
R. J. H. Scott
L. H. Walsh
L. A. Weatherly
J. Wigmore
A. S. Wohlmann

### BRIDGWATER.

F. J. C. Parsons

J. L. K. Pringle

R. H. F. Routh

### BRISLINGTON.

B. B. Fox

W. M. Morton

C. M. Phillips

### BURNHAM.

F. C. Berry

### CHEDDAR.

R. W. Statham

### CLEVEDON.

H. J. Capron
T. Davis
W. J. Hill
J. Martin

### CREWKERNE.

W. J. Penny
W. W. Webber

### CURRY RIVEL.

R. H. Vereker

### FRESHFORD.

C. E. S. Flemming

### FROME.

A. W. Dalby
J. M. Rattray
C. R. Wood

### HAM GREEN.

F. P. Mackie

### ILMINSTER.

C. Munden

### KEYNSHAM.

G. G. D. Willett

### SOMERSETSHIRE *(Continued).*

LANGPORT.

J. Morgan

LEIGH WOODS.

J. Harvey.

MILVERTON.

C. Randolph

PILL.

T. W. S. Morgan

PORTISHEAD.

G. Boyd
W. Monckton
C. A. Wigan

SOUTH PETHERTON.

S. W. MacIlwaine

TAUNTON.

H. T. S. Aveline
Taunton Hospital
H. T. Rutherford

TIMSBURY.

F. Woods

WEDMORE.

J. Ford
R. P. Tyley

WELLINGTON.

J. Meredith

WELLS.

W. Fairbanks
W. A. L. Smith

WESTON-SUPER-MARE.

H. T. M. Alford
H. S. Ballance
C. P. Crouch
G. B. Fraser
E. F. Martin
G. F. Rossiter
R. Roxburgh
J. W. Smith
G. H. Temple
J. Wallace

WORLE.

F. St. J. Kemm

WRINGTON.

H. C. Bristowe
H. W. Collins

YEOVIL.

W. R. M. Semple

### SUFFOLK.

BECCLES.

F. H. Hudson.

### SURREY.

VIRGINIA WATER.

S. R. Philipps

### WARWICKSHIRE.

LEAMINGTON.

A. W. W. Hoffman

### WILTSHIRE.

BRADFORD-ON-AVON.

W. J. A. Adye
J. Beddoe

FOVANT.

C. Clay

CALNE.

W. S. Batten

DEVIZES.

C. Eddowes

MALMESBURY.

R. Kinneir

SWINDON.

W. Howse
J. C. Maclean

WARMINSTER.

R. L. Willcox

### WORCESTERSHIRE.

WORCESTER.

R. W. Brimacombe

### Ireland.

### ANTRIM.

BELFAST.

J. W. Browne

A. Jamison

J. A. Lindsay

### Wales.

### BRECONSHIRE.

TALGARTH.

W. Howells

### CARMARTHENSHIRE.

FERRYSIDE.

P. Williams

LLANELLY.

S. J. Roderick

### CARNARVONSHIRE.

ST. CLEARS.

R. Thomas

### GLAMORGANSHIRE.

ABERDARE.

E. Jones

CARDIFF.

P. R. Griffiths
T. G. Horder
D. R. Paterson
W. Price
A. Sheen
Medical Society
J. L. Thomas
H. R. Vachell
J. Williams

GORSEINON.

T. Mitchell

MERTHYR TYDVIL.

W. W. Jones
C. E. G. Simons
J. L. W. Ward

PENARTH.

R. F. Nell

PORTH.

H. N. Davies

SWANSEA.

R. C. Elsworth
E. Le C. Lancaster

TREHARRIS.

W. W. Leigh

### PEMBROKESHIRE.

HAVERFORDWEST.

E. P. Phillips

### Belgium.

COURTRAI.

Dr. Lauwers

### Switzerland.

DAVOS-PLATZ.

W. R. Huggard

### Cape Colony.

FORT BEAUFORT.

J. Shaw

KING WILLIAM'S TOWN.

H. M. Chute

### United States.

COLORADO.

S. E. Solly

CHICAGO.

Columbus Medical Library

### Italy.

GENOA.

S. Biamonti

### Egypt.

ALEXANDRIA.

J. Mackie

CAIRO.

M. A. Ruffer

### New Zealand.

LOWER HUTT.

J. R. Purdy

### India.

BHAGELPORE.

N. Muzumider

### INDEX.

Aarons, Dr. S. J.—Golden rules of gynæcology (*Review*), 362.
Abdominal surgery, Ovariotomy and—H. Cripps (*Review*), 265.
Abdominal surgery, Some groups of interesting cases of—C. A. Morton, 203.
Achondroplasia—Dr. F. H. Edgeworth, 27; C. E. S. Flemming, 21.
Allbutt, Dr. T. C. [Ed.]—A system of medicine (*Review*), 56, 346.
American Pediatric Society, Transactions of the (*Review*), 75.
American Physicians, Transactions of the Association of (*Review*), 364.
American Surgical Association, Transactions of the (*Review*), 275.
American year-book of medicine and surgery (*Review*), 157.
Anæsthetists, The transactions of the Society of (*Review*), 277.
Anastomosis, Intestinal, 248.
Anatomy, Tablets of—T. Cooke and F. G. H. Cooke (*Review*), 54.
Anderson, Dr. T. M'C.—Contributions to clinical medicine (*Review*), 255.
Animal as a prime mover, The—R. H. Thurston (*Review*), 359.
Annual and analytical cyclopædia of practical medicine (*Reviews*), 157, 277.
Antrum of Highmore, Muco-purulent catarrh of the, 338.
Aphasia and other speech defects—Dr. H. C. Bastian (*Review*), 268.
Apoplexy, On the temperature in cases of—Dr. J. M. Clarke, 97.
Archives provinciales de médecine (*Review*), 159.
Army, Recollections of thirty-nine years in the—Sir C. A. Gordon (*Review*), 53.
Arterial disease, Ophthalmoscopic evidence of, 47.
Asthma, On so-called spasmodic—Dr. E. Kingscote (*Review*), 360.
Atkinson, Dr. T. R.—Aids to examinations (*Review*), 270.

Baby feeding (*Review*), 155.
Bacteria, Pathogenic—Dr. J. McFarland (*Review*), 272.
Bacteriology, An atlas of—Dr. C. Slater and E. J. Spitta (*Review*), 261.
Bacteriology, A manual of—Dr. R. T. Hewlett (*Review*), 353.
Bacteriology of some suppurations complicating pulmonary disease—Dr. J. O. Symes, 30.
Baldy, Dr. J. M. [Ed.]—An American text-book of gynecology (*Review*), 354.
Bangs and W. A. Hardaway, Drs. L. B. [Eds.]—An American text-book of genito-urinary diseases, syphilis, and diseases of the skin (*Review*), 64.
Bannatyne, Dr. G. A.—Rheumatoid arthritis (*Review*), 72.
Barclay, Dr. W. M.—Report on surgery, 331.
Baron, Dr. B. J.—Report on larnygology and rhinology, 336.
Bastian, Dr. H. C.—A treatise on aphasia and other speech defects (*Review*), 268.
Beard, J.—On certain problems of vertebrate embryology; The span of gestation and the cause of birth (*Reviews*), 141.
Bell, Dr. J.—Notes on surgery for nurses (*Review*), 365.
Bell, Dr. R.—Chloroform: its absolutely safe administration (*Review*), 272.
Bergey, Dr. D. H.—An investigation on the influence upon the vital resistance of animals to the micro-organisms of disease brought about by prolonged sojourn in an impure atmosphere (*Review*), 272.
Bicêtre—Recherches cliniques et thérapeutiques sur l'épilepsie, l'hystérie, et l'idiotie (*Review*), 156.
Birth, The span of gestation and the cause of—J. Beard (*Review*), 141.
Blake, Dr. E.—On the study of the hand for indications of local and general disease (*Review*), 359.
Blood, The—Dr. A. C. Coles (*Review*), 55.
Boston Society of Medical Sciences, Journal of the (*Review*), 77.
Bradycardia, A case of extreme—R. G. Fendick and Dr. G. Parker, 113; Notes on the eye symptoms—F. R. Cross, 113.
Breast, Cancer of the, 128.

Breast, Diseases of the—Dr. A. M. Sheild (*Review*), 266.
Breast, Extensive operations for cancer of the, 331.
Bristol in the eighteenth century, Medical—W. H. Harsant, 297.
Bristol Medico-Chirurgical Society, 79, 161, 367.
British food journal, The (*Review*), 159.
British physician, The (*Review*), 278.
Brodie, Sir Benjamin Collins—T. Holmes (*Review*), 140.
Brodie, Dr. T. G.—The essentials of experimental physiology (*Review*), 54.
Bronchitis, On the treatment of sub-acute—Dr. F. H. Edgeworth, 197.
Burghard, Drs. W. W. Cheyne and F. F.—A manual of surgical treatment (*Review*), 350.
Burr, Dr. C. B.—A primer of psychology and mental diseases (*Review*), 357.
Bush, J. P.—Some unusual operations on lunatics and their results, 219.

Caledonian medical journal (*Review*), 278.
Cancer and tuberculosis, The ferment treatment of—Dr. H. Manders (*Review*), 262.
Cancer of the breast, 128.
Cancer of the tongue, 130.
Cardio-vascular repair, Observations on—Dr. W. B. Thorne (*Review*), 360.
Carless, W. Rose and A.—A manual of surgery (*Review*), 143.
Carpus backwards, A case of dislocation of the hand and—Dr. T. Mitchell, 121.
Carwardine, T.—Report on surgery, 244.
Case paper—Drs. J. K. Crouch and E. Le C. Lancaster (*Review*), 365.
Cataract, A treatise on "unripe"—Dr. W. A. M'Keown (*Review*), 356.
Cataract extraction, 46.
Catheterisation of the ureters, 244.
Cautley, Dr. E.—The natural and artificial methods of feeding infants and young children (*Review*), 69.
Cerebro-spinal meningitis, Epidemic—Drs. W. T. Councilman, F. B. Mallory, and J. H. Wright (*Review*), 270.
Chavasse's Advice to a mother (*Review*), 73; Advice to a wife (*Review*), 73.
Chemistry, Practical organic—S. Rideal (*Review*), 154.
Cheyne and F. F. Burghard, Drs. W. W.—A manual of surgical treatment (*Review*), 350.
Childhood, Medical diseases of infancy and—Dr. D. Williams (*Review*), 151.
Children, An American text-book of the diseases of—Dr. L. Starr [Ed.] (*Review*), 355.
Children, Clinical examination and treatment of sick—Dr. J. Thomson (*Review*), 151.
Children, Dementia in, 330.
Children, General paresis in, 329.
Children, Lumbar puncture in, 326.
Children, Nervous diseases following acute specific fevers in, 328.
Children, Pocket formulary for the treatment of disease in—Dr. L. Freyberger (*Review*), 363.
Children, The study of—Dr. F. Warner (*Review*), 69.
Children's teeth, The diseases of—R. D. Pedley (*Review*), 74.
Chirurgie, Association Française de. 11e Congrès. Procès-verbaux (*Review*), 156.
Chloroform—Dr. R. Bell (*Review*), 272.
Chreiman, M. A.—Health loss and gain (*Review*), 155.
Clarke, Dr. J. M.—On the temperature in cases of apoplexy, and on the occurrence (1) of œdema and (2) loss of the knee-jerk in the paralysed limbs in hemiplegia, 97; Report on medicine, 326.
Climatology, Medical—Dr. S. E. Solly (*Review*), 145.
Clinical investigation, Atlas of methods of—Dr. C. Jakob (*Review*), 142.
Clinical Society of London, Transactions of the (*Review*), 274.
Clouston, Dr. T. S. — Clinical lectures on mental diseases (*Review*), 258.
Coles, Dr. A. C.—The blood: how to examine and diagnose its diseases (*Review*), 55.
Consumptive sanatorium for the people, The erection of a—Dr. Nahm (*Review*), 362.
Consumptives, Sanatoria for—Dr. F. R. Walters (*Review*), 348.
Cooke, T. and F. G. H.—Tablets of anatomy (*Review*), 54.

Corfield, Dr. W. H. — Dwelling houses: their sanitary construction and arrangement (*Review*), 155.
Cory, Dr. R. — Lectures on the theory and practice of vaccination (*Review*), 153.
Cotton, Dr. W.—A simple form of influence machine for X-ray work, 222.
Councilman, F. B. Mallory, and J. H. Wright, Drs. W. T.—Epidemic cerebro-spinal meningitis and its relation to other forms of meningitis (*Review*), 270.
Crile, Dr. G. W.—An experimental research into surgical shock (*Review*), 352.
Cripps, H.—Ovariotomy and abdominal surgery (*Review*), 265.
Cross, F. R.—Notes on the eye-symptoms of a case of extreme bradycardia, 113.

DaCosta, Dr. J. C.—A manual of modern surgery (*Review*), 259.
Darwin and the theory of natural selection, Charles—E. B. Poulton (*Review*), 254.
Dementia in the young, 330.
Dermatitis, 249.
Dermatological Society of Great Britain and Ireland, Transactions of the (*Review*), 76.
Dermatology, Report on—Dr. H. Waldo, 249.
Devon and Exeter Medico-Chirurgical Society, 175.
Diabetes mellitus and its treatment —Dr. R. T. Williamson (*Review*), 60.
(Diabetes mellitus), Wesen, ursache und behandlung der zuckerkrankheit—Dr. A. Lenné (*Review*), 61.
Diagnosis, A clinical text-book of medical—Dr. O. Vierordt (*Review*), 344.
Diarrhœa, Treatment of summer—Irrigation of the large intestine, 339; Lavage of the stomach, 340.
Dictionary, Lippincott's medical (*Review*), 138.
Dictionary of medical terms (English-French)—H. de Méric (*Review*), 342.
Diet and food—Dr. A. Haig (*Review*), 269.
Diet in chronic kidney disease, 244.
Diphtheria, Epidemic—Dr. A. Newsholme (*Review*), 70.
Diphtheria, Vomiting and cardiac failure in connection with, 339.
Directory of booksellers and bibliophile's manual, The international (*Review*), 366.
Dislocation of the hand and carpus backwards, A case of—Dr. T. Mitchell, 121.
Dispensing for nurses, Notes on pharmacy and—C. J. S. Thompson (*Review*), 155.
Dowse, Dr. T. S.—The treatment of disease by physical methods (*Review*), 361.
Dwelling houses—Dr. W. H. Corfield (*Review*), 155.

Ear, Diseases of the—Dr. E. B. Gleason (*Review*), 273.
Edgeworth, Dr. F. H.—Achondroplasia, 27; On the treatment of subacute bronchitis, 197.
Edinburgh medical journal (*Reviews*), 158, 278.
Edinburgh Obstetrical Society, Transactions of the (*Reviews*), 276, 365.
Edkins, Drs. E. Klein and J. S.—Elements of histology (*Review*), 154.
Electro-therapeutics, Conservative gynecology and—Dr. G. B Massey (*Review*), 150.
Embolism of the mesenteric vessels, 41.
Embryology, On certain problems of vertebrate—J. Beard (*Review*), 141.
Encyclopædia medica—Dr. C. Watson [Ed.] (*Review*), 344.
Endocarditis, A case of acute ulcerative—Dr. H. L. Ormerod, 15.
English sanitary institutions—Sir J. Simon (*Review*), 138.
Épilepsie, l'hystérie et l'idiotie, Recherches cliniques et thérapeutiques sur l' (*Review*), 156.
Epiphyses, Traumatic separation of the—J. Poland (*Review*), 260.
Examinations, Aids to—Dr. T. R. Atkinson (*Review*), 270.
Eyeball, Excision of the, 48
Eyeball, Use of electro-magnet in extracting foreign bodies in the, 45.
Eye-reflexes in infants, Condition of the pupils and, 327.
Eye, System of diseases of the—Drs. W. F. Norris and C. A. Oliver [Eds.] (*Review*), 267.

Fayrer, Sir J.—Inspector-General Sir James Ranald Martin (*Review*), 50.

Feeding infants and young children, The natural and artificial methods of—Dr. E. Cautley (*Review*), 69.
Fendick and Dr. G. Parker, R. G.—A case of extreme bradycardia, 113.
Fenwick, E. H.—Golden rules of surgical practice (*Review*), 361.
Ferment treatment of cancer and tuberculosis, The—Dr. H. Manders (*Review*), 262.
Filters, An inquiry into the relative efficiency of water—Drs. G. S. Woodhead and G. E. C. Wood (*Review*), 71.
Firth, J. L.—Report on surgery, 41.
Fisher, Dr. T.—Report on medicine, 36.
Flemming, C. E. S. — Achondroplasia, 21.
Forensic medicine and toxicology—Dr. J. D. Mann (*Review*), 70.
Formulæ, Physicians' book for private (*Review*), 280.
Freyberger, Dr. L. — The pocket formulary for the treatment of disease in children (*Review*), 363.
From our dead selves to higher things—F. J. Gant (*Review*), 72.
Frontal sinus, Chronic empyema of the, 337.
Fürbringer, Dr. P.—Text-book of diseases of the kidneys and genito-urinary organs (*Review*), 65.
Fürstner, Dr. C. — Wie ist die fürsorge für gemüthskranke von aerzten und laien zu fördern? (*Review*), 270.

Gadd, H. W.—A synopsis of the British Pharmacopœia, 1898 (*Review*), 272.
Gant, F. J.—From our dead selves to higher things (*Review*), 72.
Gastric ulcer, 238.
Gemüthskranke von aerzten und laien zu fördern, Wie ist die fürsorge für—Dr. C. Fürstner (*Review*), 270.
General paresis in the young, 329.
Genito-urinary diseases, syphilis, and diseases of the skin, An American text-book of—Drs. L. B. Bangs and W. A. Hardaway [Eds.] (*Review*), 64.
Genito-urinary surgery and venereal diseases—Drs. J. W. White and E. Martin (*Review*), 144.
Gestation and the cause of birth, The span of—J. Beard (*Review*), 141.
Gibson, Dr. G. A.—Diseases of the heart and aorta (*Review*), 257.
Glands, The surgical anatomy of the lymphatic—Dr. C. H. Leaf (*Review*), 349.
Glasses for prevention of eye injuries, Protective, 46.
Gleason, Dr. E. B.—Essentials of the diseases of the ear (*Review*), 273.
Glycerin, The properties and uses of pure—M.D. (*Review*), 362.
Gordon, Sir C. A.—Recollections of thirty-nine years in the army (*Review*), 53.
Gowers, Sir W. R.—A manual of diseases of the nervous system (*Review*), 347.
Grant College Medical Society Transactions of the (*Review*), 274.
Grèce médicale, La (*Review*), 158.
Greene, R.—The Schott treatment for chronic heart diseases (*Review*), 271.
Grünwald, Dr. L.—Atlas and abstract of the diseases of the larynx (*Review*), 357.
Gynæcology, Golden rules of—Dr. S. J. Aarons (*Review*), 362.
Gynæcology, Report on—Dr. W. C. Swayne, 131.
Gynecology, An American text-book of—Dr. J. M. Baldy [Ed.] (*Review*), 354.
Gynecology and electro-therapeutics, Conservative—Dr. G. B. Massey (*Review*), 150.
Gynecology, Operative—Dr. H. A. Kelly (*Review*), 263.

Haig, Dr. A.—Diet and food, considered in relation to strength and power of endurance, training and athletics (*Review*), 269.
Halliburton, Dr. W. D.—Kirkes' hand-book of physiology (*Review*), 343.
Hallucinations and illusions — E. Parish (*Review*), 55.
Hand and carpus backwards, A case of dislocation of the — Dr. T. Mitchell, 121.
Hand for indications of local and general disease, On the study of the—Dr. E. Blake (*Review*), 359.
Hardaway, Drs. L. B. Bangs and W. A. [Eds.]—An American text-book of genito-urinary diseases, syphilis, and diseases of the skin (*Review*), 64.
Hare, Dr. F. E.—The cold-bath treatment of typhoid fever (*Review*), 258.
Hare, Dr. H. A.—A text-book of practical therapeutics (*Review*), 62.

Harley, George—Mrs. A. Tweedie [Ed.] (*Review*), 342.
Harsant, W. H.—Medical Bristol in the eighteenth century, 297.
Headache, Nasal and aural disease as a cause of, 336.
Health loss and gain.—M. A. Chreiman (*Review*), 155.
Health resorts of Europe, The mineral waters and—Dr. H. and F. P. Weber (*Review*), 146.
Heart and aorta, Diseases of the —Dr. G. A. Gibson (*Review*), 257.
Heart diseases, The Schott treatment for chronic—R. Greene (*Review*), 271.
Hemiplegia, On the occurrence (1) of œdema, and (2) of loss of the knee-jerk in the paralysed limbs in—Dr. J. M. Clarke, 97.
Hemorrhage, Post-partum, 131.
Herz, Dr. H.—Die störungen des verdauungsapparates als ursache und folge anderer erkrankungen (*Review*), 255.
Hewlett, Dr. R. T.—A manual of bacteriology (*Review*), 353.
Histology, Elements of—Drs. E. Klein and J. S. Edkins (*Review*), 154.
Hofmann, Dr. E. von—Atlas of legal medicine (*Review*), 358.
Holmes, T.—Sir Benjamin Collins Brodie (*Review*), 140.
Hospitals and charities, Burdett's (*Review*), 365.
Hurst, Drs. A. M. Marshall and C. H.—A junior course of practical zoology (*Review*), 343.
Hysterectomy for fibroid tumour of uterus—Dr. W. H. C. Newnham, 8.

'Ιατρική Πρόοδος (*Review*), 158.
Illusions, Hallucinations and—E. Parish (*Review*), 55.
Index-catalogue of the library of the Surgeon-General's Office, United States Army (*Review*), 76.
Index medicus, The, 189.
Infancy and childhood, Medical diseases of—Dr. D. Williams (*Review*), 151.
Infancy and childhood, Therapeutics of—Dr. A. Jacobi (*Review*), 68.
Infants and young children, The natural and artificial methods of feeding—Dr. E. Cautley (*Review*), 69.
Infants, Condition of the pupils and eye-reflexes in, 327.
Influence machine for X-ray work, A simple form of—Dr. W. Cotton, 222.
Intestinal anastomosis, 248.
Intussusception, On irreducible—Dr. G. L. K. Pringle, 322.
Iowa State Medical Society, Transactions of the (*Review*), 274.
Ireland, Transactions of the Royal Academy of Medicine in (*Review*), 274.
Irrigation of the large intestine in summer diarrhœa, 339.

Jacobi, Dr. A.—Therapeutics of infancy and childhood (*Review*), 68.
Jakob, Dr. C.—Atlas of methods of clinical investigation, with an epitome of clinical diagnosis and of special pathology and treatment of internal diseases (*Review*), 142.
Jellett, Dr. H.—A short practice of midwifery (*Review*), 149.
Johns Hopkins hospital reports (*Review*), 74.
Journal of the Boston Society of Medical Sciences (*Review*), 77.
Journal of tropical medicine, The (*Review*), 77.

Keen, Dr. W. W.—The surgical complications and sequels of typhoid fever (*Review*), 351.
Kelly, Dr. H. A.—Operative gynecology (*Review*), 263.
Kelynack, Dr. T. N.—Renal growths (*Review*), 145.
Kidney disease, Diet in chronic, 244.
Kidney, Rupture of the, 44.
Kidneys and genito-urinary organs, Diseases of the—Dr. P. Fürbringer (*Review*), 65.
King's College hospital reports (*Review*), 156.
Kingscote, Dr. E.—On so-called spasmodic asthma (*Review*), 360.
Kirkes' hand-book of physiology—Dr. W. D. Halliburton (*Review*), 343.
Klein and J. S. Edkins, Drs. E.—Elements of histology (*Review*), 154.
Knee-jerk in the paralysed limbs in hemiplegia, Loss of—Dr. J. M. Clarke, 97.
Knowledge (*Review*), 279.

Laryngology, Report on—Dr. B. J. Baron, 336.

Larynx, Atlas and abstract of the diseases of the—Dr. L. Grünwald (*Review*), 357.
Lazarus - Barlow, Dr. W. S.—A manual of general pathology (*Review*), 147.
Leaf, Dr. C. H.—The surgical anatomy of the lymphatic glands (*Review*), 349.
Legal medicine, Atlas of—Dr. E. von Hofmann (*Review*), 358.
Lenné, Dr. A.—Wesen, ursache und behandlung der zuckerkrankheit (Diabetes mellitus) (*Review*), 61.
Library of the Bristol Medico-Chirurgical Society, 89, 177, 282, 370.
Liebig, Justus von—W. A. Shenstone (*Review*), 140.
Lippincott's medical dictionary (*Review*), 138.
Liver, Operative treatment of cirrhosis of the, 247.
Local medical notes, 92, 181, 287, 374.
London, Transactions of the Clinical Society of (*Review*), 274.
Loomis and W. G. Thompson, Drs. A. L. [Eds.] — A system of practical medicine by American authors (*Review*), 58.
Lumbar puncture in children, 326.
Lunatics, Some unusual operations on—J. P. Bush, 219.

McFarland, Dr. J.—A text-book upon the pathogenic bacteria (*Review*), 272.
M'Keown, Dr. W. A.—A treatise on "unripe" cataract (*Review*), 356.
Maddox, Dr. E. E.—Tests and studies of the ocular muscles (*Review*), 152.
Malaria, 236.
Malaria, Notes on—Surgeon-Major E. M. Wilson (*Review*), 154.
Malarial fevers, Lectures on the—Dr. W. S. Thayer (*Review*), 59.
Mallory, and J. H. Wright, Drs. W. T. Councilman, F. B.—Epidemic cerebro-spinal meningitis and its relation to other forms of meningitis (*Review*), 270.
Manders, Dr. H. — The ferment treatment of cancer and tuberculosis (*Review*), 262.
Mann, Dr. J. D.—Forensic medicine and toxicology (*Review*), 70.
Manson, Dr. P.—Tropical diseases (*Review*), 260.
Marshall and C. H. Hurst, Drs. A. M.—A junior course of practical zoology (*Review*), 343.
Martin, Drs. J. W. White and E.—Genito-urinary surgery and venereal diseases (*Review*), 144.
Martin, Inspector-General Sir James Ranald—Sir J. Fayrer (*Review*), 50.
Martindale, W.—Drs. Harvey and Davidson's syllabus of materia medica (*Review*), 361.
Martindale and Dr. W. W. Westcott, W.—The extra pharmacopœia (*Review*), 73.
Massey, Dr. G. B.—Conservative gynecology and electro-therapeutics (*Review*), 150.
Materia medica, Drs. Harvey and Davidson's syllabus of—W. Martindale (*Review*), 361.
Materia medica, pharmacy, pharmacology, and therapeutics — Dr. W. H. White (*Review*), 155.
Medical annual (*Review*), 158.
Medical Council, Minutes of the General (*Review*), 270.
Medical defence, A year's work in (*Review*), 278.
Medical Society of London, Transactions of the (*Review*), 364.
Medicine, A system of—Dr. T. C. Allbutt [Ed.] (*Reviews*), 56, 346.
Medicine, A system of practical, by American authors — Drs. A. L. Loomis and W. G. Thompson [Eds.] (*Review*), 58.
Medicine, Contributions to clinical —Dr. T. M'C. Anderson (*Review*), 255.
Medicine, History of—Dr. R. Park (*Review*), 50.
Medicine, Practice of—Dr. F. Taylor (*Review*), 255.
Medicine, Reports on—Dr. J. M. Clarke, 326; Dr. T. Fisher, 35; Dr. G. Parker, 236; Dr. R. S. Smith, 121.
Medico - chirurgical transactions (*Review*), 363.
Mental diseases—Dr. T. S. Clouston (*Review*), 258.
Merck's annual report (*Review*), 277.
Méric, H. de—Dictionary of medical terms (English-French) (*Review*), 342.
Mesenteric vessels, Thrombosis and embolism of the, 41.
Mexico—Informes rendidos por los inspectores sanitarios de cuartel y por los de los distritos al consejo superior de salubridad (*Review*), 363.

Michigan State Medical Society, Transactions of the (*Review*), 364.
Microbes, The war with the—E. A. de Schweinitz (*Review*), 362.
Middlesex hospital reports (*Review*), 74.
Midwifery—Dr. H. Jellett (*Review*), 149; Dr. W. S. Playfair (*Review*), 354; Dr. D. L. Roberts (*Review*), 67.
Mineral waters and health resorts of Europe—Drs. H. and F. P. Weber (*Review*), 146.
Mitchell, Dr. T.—A case of dislocation of the hand and carpus backwards, 121.
Moore, Dr. J. E.—Orthopedic surgery (*Review*), 67.
Morris, H.—On the origin and progress of renal surgery (*Review*), 259.
Morris, M.—Diseases of the skin (*Review*), 273.
Morton, C. A.—Report on surgery, 126; Some groups of interesting cases of abdominal surgery, 203.
Mother, Chavasse's Advice to a (*Review*), 73.
Muscular atrophy, A case of neuritic—Dr. J. E. Shaw, 319.
Myoma of the uterus, The surgical treatment of—W. R. Williams, 1.

Nahm, Dr.—The erection of a consumptive sanatorium for the people (*Review*), 362.
Nasal obstruction—W. J. Walsham (*Review*), 152.
Nervensystems, Pathologie und pathologischen anatomie des central- —Dr. A. Pick (*Review*), 148.
Nervous diseases following acute specific fevers in children, 328.
Nervous system, A manual of diseases of the—Sir W. R. Gowers (*Review*), 347.
Newnham, Dr. W. H. C.—A case of hysterectomy for fibroid tumour of the uterus, with intra-abdominal treatment of pedicle, 8.
Newsholme, Dr. A.—Epidemic diphtheria; a research on the origin and spread of the disease from an international standpoint (*Review*), 70.
New York, Transactions of the Medical Society of the State of (*Review*), 275.
Norris and C. A. Oliver, Drs. W. F. [Eds.]—System of diseases of the eye (*Review*), 267.
Nurses, Notes on pharmacy and dispensing for—C. J. S. Thompson (*Review*), 155.
Nursing directory, Burdett's official (*Review*), 279.

Obstetrics, Report on—Dr. W. C. Swayne, 131.
Ocular muscles, The—Dr. E. E. Maddox (*Review*), 152.
O'Dwyer, Memorial to Dr. Joseph, 189.
Œdema in the paralysed limbs in hemiplegia—Dr. J. M. Clarke, 97.
Ohio State Medical Society, Transactions of the (*Review*), 275.
Oliver, Drs. W. F. Norris and C. A. [Eds.]—System of diseases of the eye (*Review*), 267.
Ophthalmia neonatorum, 49.
Ophthalmological Society of the United Kingdom, Transactions of the (*Review*), 276.
Ophthalmology, Report on—C. H. Walker, 45.
Ormerod, Dr. H. L.—A case of acute ulcerative endocarditis, 15.
Orthopedic surgery—Dr. J. E. Moore (*Review*), 67; Dr. W. M. Shaffer (*Review*), 74.
Ovariotomy and abdominal surgery—H. Cripps (*Review*), 265.

Paresis in the young, General, 329.
Parish, E.—Hallucinations and illusions (*Review*), 55.
Park, Dr. R.—An epitome of the history of medicine (*Review*), 50.
Parker, Dr. G.—Report on medicine, 236.
Parker and R. G. Fendick, Dr. G.—A case of extreme bradycardia, 113.
Patella, Fracture of the—A. W. Prichard, 104.
Pathology, General—Dr. W. S. Lazarus-Barlow (*Review*), 147.
Pedley, R. D.—The diseases of children's teeth (*Review*), 74.
Penrose, Dr. C. B.—A text-book of diseases of women (*Review*), 150.
Peritoneum after severe injuries, Tolerance of the, 248.
Pharmacopœia, 1898, A synopsis of the British—H. W. Gadd (*Review*), 272.
Pharmacopœia, The extra—W. Martindale and Dr. W. W. Westcott (*Review*), 73.
Pharmacy and dispensing for nurses, Notes on—C. J. S. Thompson (*Review*), 155.
Philadelphia monthly medical journal, The (*Review*), 159.
Philadelphia, The College of Physicians of, 381.

Philadelphia, Transactions of the College of Physicians of (*Review*), 75.
Physiology, Kirkes' handbook of—Dr. W. D. Halliburton (*Review*), 343.
Physiology, The essentials of experimental—Dr. T. G. Brodie (*Review*), 54.
Pick, Dr. A.—Beiträge zur pathologie und pathologischen anatomie des central nervensystems (*Review*), 148
Pityriasis rosea, 251.
Pityriasis rubra, 249.
Playfair, Dr. W. S.—A treatise on the science and practice of midwifery (*Review*), 354.
Poland J.—Traumatic separation of the epiphyses (*Review*), 260.
Poliomyelitis, Causes of acute, 329.
Polyclinic, The (*Review*), 160.
Poulton, E. B.—Charles Darwin and the theory of natural selection (*Review*), 254.
Prichard, A. W.—Fracture of the patella, 104.
Pringle, Dr. G. L. K.—Irreducible intussusception: with notes on a fatal case, 322.
Psilosis or "sprue"—Dr. G. Thin (*Review*), 143.
Psychology and mental disease, A primer of—Dr. C. B. Burr (*Review*), 357.
Pterygium, Etiology of, 47.
Purdy, Dr. C. W.—Practical uranalysis and urinary diagnosis (*Review*), 359.

Renal growths—Dr. T. N. Kelynack (*Review*), 145.
Renal surgery—H. Morris (*Review*), 259.
Renal tuberculosis, 239.
Respiratory tract, Diseases of the upper—Dr. P. W. Williams (*Review*), 74.
Retinitis proliferans, 48.
Rheumatoid arthritis—Dr. G. A. Bannatyne (*Review*), 72.
Rhinology, Report on—Dr. B. J. Baron, 336.
Rideal, S.—Practical organic chemistry (*Review*), 154.
Roberts, Dr. D. L.—The practice of midwifery (*Review*), 67.
Roentgen ray, Archives of the (*Review*), 278; A simple form of influence machine for X-ray work—Dr. W. Cotton, 222; X-rays in ophthalmology, 46.
Rogers, Dr. B. M. H.—Idiopathic thrombosis of the portal and contributory veins, 107; Report on therapeutics, 339.
Rose and A. Carless, W.—A manual of surgery (*Review*), 143.
Rotunda hospitals, Clinical report of the (*Review*), 75.

Saint Bartholomew's hospital reports (*Review*), 157.
Saint Thomas's hospital reports (*Review*), 365.
Sanatoria for consumptives — Dr. F. R. Walters (*Review*), 348.
Sanatorium for the people, The erection of a consumptive—Dr. Nahm (*Review*), 362.
Sanitary institutions, English—Sir J. Simon (*Review*), 138.
Schenk, Dr. L.—The determination of sex (*Review*), 72.
Schott treatment for chronic heart diseases, The—R. Greene (*Review*), 271.
Schweinitz, E. A. de—The war with the microbes (*Review*), 362.
Scraps, 93, 190, 295, 381.
Sex, The determination of—Dr. L. Schenk (*Review*), 72.
Shaffer, Dr. N. M.—Brief essays on orthopædic surgery (*Review*), 74.
Shaw, Dr. J. E.—A case of neuritic muscular atrophy ("peroneal" type), 319.
Sheild, Dr. A. M.—A clinical treatise on diseases of the breast (*Review*), 266.
Shenstone, W. A.—Justus von Liebig: his life and work (*Review*), 140.
Shock, An experimental research into surgical—Dr. G. W. Crile (*Review*), 352.
Sick, Preparations for the, 77, 160, 280, 366.
Simon, Sir J.—English sanitary institutions (*Review*), 138.
Skene, Dr. A. J. C.—Treatise on the diseases of women (*Review*), 68.
Skin, An American text-book of genito-urinary diseases, syphilis, and diseases of the—Drs. L. B. Bangs and W. A. Hardaway [Eds.] (*Review*), 64.
Skin, Diseases of the—M. Morris (*Review*), 273.
Slater and E. J. Spitta, Dr. C.—An atlas of bacteriology (*Review*), 261.
Smith, Dr. R. S.—Report on medicine, 121.
Soap, The antiseptic and disinfectant properties of—Dr. J. O. Symes, 193.
Societies: Bristol Medico-Chirurgical, 79, 161, 367; Devon and Exeter Medico-Chirurgical, 175.

Solly, Dr. S. E.—A handbook of medical climatology (*Review*), 145.
South Carolina Medical Association, Transactions of the (*Reviews*), 275, 364.
Spencer, W. G.—Outlines of practical surgery (*Review*), 62.
Spitta, Dr. C. Slater and E. J.—An atlas of bacteriology (*Review*), 261.
Starr, Dr. L. [Ed.]—An American text-book of the diseases of children (*Review*), 355.
Stevenson, Surgeon-Colonel W. F.—Wounds in war (*Review*), 63.
Stewart, Dr. R. W.—The diseases of the male urethra (*Review*), 66.
Stroebe, Dr. H.—Ueber die wirkung des neuen tuberkulins TR auf gewebe und tuberkelbacillen (*Review*), 261.
Suppurations complicating pulmonary disease, The bacteriology of some—Dr. J. O. Symes, 30.
Surgery, Atlas and epitome of operative—Dr. O. Zuckerkandl (*Review*), 351.
Surgery—Dr. J. C. DaCosta (*Review*), 259; W. Rose and A. Carless (*Review*), 143; R. F. Tobin (*Review*), 272.
Surgery for nurses, Notes on—Dr. J. Bell (*Review*), 365.
Surgery, Practical—W. G. Spencer (*Review*), 62.
Surgery, Reports on—W. M. Barclay, 331; T. Carwardine, 244; J. L. Firth, 41; C. A. Morton, 126.
Surgical practice, Golden rules of—E. H. Fenwick (*Review*), 361.
Surgical treatment, A manual of—Drs. W. W. Cheyne and F. F. Burghard (*Review*), 350.
Swayne, Dr. W. C.—Report on obstetrics and gynæcology, 131; Symphysiotomy, 11.
Symes, Dr. J. O.—The antiseptic and disinfectant properties of soap, 193; The bacteriology of some suppurations complicating pulmonary disease, 30.
Symphysiotomy—Dr. W. C. Swayne, 11.
Syphilis and diseases of the skin, An American text-book of genito-urinary diseases—Drs. L. B. Bangs and W. A. Hardaway [Eds.] (*Review*), 64.

Taylor, Dr. F.—A manual of the practice of medicine (*Review*), 255.
Teeth, The diseases of children's—R. D. Pedley (*Review*), 74.
Temperature in cases of apoplexy, On the—Dr. J. M. Clarke, 97.
Thayer, Dr. W. S.—Lectures on the malarial fevers (*Review*), 59.
Therapeutic notes (*Review*), 278.
Therapeutics of infancy and childhood—Dr. A. Jacobi (*Review*), 68.
Therapeutics, Practical—Dr. H. A. Hare (*Review*), 62.
Therapeutics, Report on—Dr. B. M. H. Rogers, 339.
Thin, Dr. G.—Psilosis or "sprue" (*Review*), 143.
Thompson, C. J. S.—Notes on pharmacy and dispensing for nurses (*Review*), 155.
Thompson, Drs. A. L. Loomis and W. G. [Eds.]—A system of practical medicine by American authors (*Review*), 58.
Thomson, Dr. J.—Guide to the clinical examination and treatment of sick children (*Review*), 151.
Thorne, Dr. W. B.—Observations on cardio-vascular repair (*Review*), 360.
Thrombosis of the mesenteric vessels, 41.
Thrombosis of the portal and contributory veins, Idiopathic—Dr. B. M. H. Rogers, 107.
Thurston, R. H.—The animal as a prime mover (*Review*), 359.
Tobin, R. F.—A synopsis of surgery (*Review*), 272.
Tongue, Cancer of the, 130.
Tonsillitis, Acute, 339.
Toxicology, Forensic medicine and—Dr. J. D. Mann (*Review*), 70.
Treatment of disease by physical methods—Dr. T. S. Dowse (*Review*), 361.
Tropical diseases—Dr. P. Manson (*Review*), 260.
Tropical medicine, The journal of (*Review*), 76.
Tubercle, How to avoid—Dr. A. T. Wise (*Review*), 271.
Tuberculosis, 121.
Tuberculosis (*Review*), 365.
Tuberculosis, The ferment treatment of cancer and—Dr. H. Manders (*Review*), 262.
Tuberculosis, The treatment and prevention of, 36.
Tuberkulins TR, Ueber die wirkung des neuen—Dr. H. Stroebe (*Review*), 261.
Tweedie, Mrs. A. [Ed.]—George Harley (*Review*), 342.

Typhoid fever, The cold-bath treatment of—Dr. F. E. Hare (*Review*), 258.
Typhoid fever, The surgical complications of—Dr. W. W. Keen (*Review*), 351.

Ulcerative endocarditis, A case of acute—Dr. H. L. Ormerod, 15.
Uræmia, 243.
Uranalysis, Practical—Dr. C. W. Purdy (*Review*), 359.
Ureters, Catheterisation of the, 244.
Urethra, The diseases of the male—Dr. R. W. Stewart (*Review*), 66.
Uterine fibroids, Treatment of the pedicle after the removal of, 126.
Uterine neoplasms, The varieties of —W. R. Williams, 314.
Uterus, Malignant disease of the, 133.
Uterus, The surgical treatment of myoma of the—W. R. Williams, 1.

Vaccination, The theory and practice of—Dr. R. Cory (*Review*), 153.
Venereal diseases, Genito-urinary surgery and—Drs. J. W. White and E. Martin (*Review*), 144.
Verdauungsapparates, Die störungen des—Dr. H. Herz (*Review*), 255.
Vierordt, Dr. O.—A clinical text-book of medical diagnosis (*Review*), 344.
Viscera, Air-inflation of the hollow, 246.
Vital resistance of animals to the micro-organisms of disease brought about by prolonged sojourn in an impure atmosphere, An investigation on the influence upon the—Dr. D. H. Bergey (*Review*) 272.

Waldo, Dr. H.—Report on dermatology, 249.
Walker, C. H.—Report on ophthalmology, 45.
Walsham, W. J.—Nasal obstruction (*Review*), 152.
Walters, Dr. F. R.—Sanatoria for consumptives (*Review*), 348.
War, Wounds in—Surgeon-Colonel W. F. Stevenson (*Review*), 63.
Warner, Dr. F.—The study of children, and their school training (*Review*), 69.
Watson, Dr. C. [Ed.]—Encyclopædia medica (*Review*), 344.
Weber, Drs. H. and F. P.—The mineral waters and health resorts of Europe (*Review*), 146.
Wellcome club and institute, Programme of the inauguration of the (*Review*), 280.
Westcott, W. Martindale and Dr. W. W.—The extra pharmacopœia (*Review*), 73.
White and E. Martin, Drs. J. W. — Genito-urinary surgery and venereal diseases (*Review*), 144.
White, Dr. W. H.—Materia medica, pharmacy, pharmacology and therapeutics (*Review*), 155.
Wife, Chavasse's Advice to a (*Review*), 73.
Williams, Dr. D.—Medical diseases of infancy and childhood (*Review*), 151.
Williams, Dr. P. W.—Diseases of the upper respiratory tract (*Review*), 74.
Williams, W. R.—The surgical treatment of myoma of the uterus, 1; The varieties of uterine neoplasms and their relative frequency, 314.
Williamson, Dr. R. T.—Diabetes mellitus and its treatment (*Review*), 60.
Wilson, Surgeon-Major E. M.—Notes on malaria, in connection with meteorological conditions at Sierra Leone (*Review*), 154.
Wise, Dr. A. T.—How to avoid tubercle (*Review*), 271.
Women, Diseases of—Dr. C. B. Penrose (*Review*), 150; Dr. A. J. C. Skene (*Review*), 68.
Wood, Drs. G. S. Woodhead and G. E. C.—An inquiry into the relative efficiency of water filters in the prevention of infective disease (*Review*), 71.
Woodhead and G. E. C. Wood, Dr. G. S.—An inquiry into the relative efficiency of water filters in the prevention of infective disease (*Review*), 71.
Wright, Drs. W. T. Councilman, F. B. Mallory, and J. H.—Epidemic cerebro-spinal meningitis and its relation to other forms of meningitis (*Review*), 270.

X-rays, *see* Roentgen ray.

Year-book of scientific and learned societies (*Review*), 279.
Year-book of treatment (*Review*), 76.

Zoology, Practical—Drs. A. M. Marshall and C. H. Hurst (*Review*), 343.
Zuckerkandl, Dr. O.—Atlas and epitome of operative surgery (*Review*), 351.

